# Data analysis of polygalacturonase inhibiting proteins (PGIPs) from agriculturally important proteomes

**DOI:** 10.1016/j.dib.2023.109831

**Published:** 2023-11-19

**Authors:** Sudha Acharya, Hallie A. Troell, Rebecca L. Billingsley, Katherine S. Lawrence, Daniel S. McKirgan, Nadim W. Alkharouf, Vincent P. Klink

**Affiliations:** a Department of Computer and Information Sciences, Towson University, Towson, MD 21252, United States; b USDA-ARS-NEA-BARC Molecular Plant Pathology Laboratory Building 004, Room 122, BARC-West, 10300 Baltimore Ave., Beltsville, MD 20705, United States; c Department of Biological Sciences, Mississippi State University, Mississippi State, MS 39762, United States; d Department of Biochemistry, Molecular Biology, Entomology and Plant Pathology, Mississippi State University, Mississippi State, MS 39762, United States; e Department of Entomology and Plant Pathology, Auburn University, 209 Life Science Building, Auburn, AL 36849, United States

**Keywords:** Plant interactions, Polygalacturonase inhibiting protein (PGIP), Soybean, *Heterodera glycines*, *Beta vulgaris*, Sugar beet

## Abstract

The plant cell wall structure can be altered by pathogen-secreted polygalacturonases (PGs) that cleave the α-(1→4) linkages occurring between D-galacturonic acid residues in homogalacturonan. The activity of the PGs leads to cell wall maceration, facilitating infection. Plant PG inhibiting proteins (PGIPs) impede pathogen PGs, impairing infection and leading to the ability of the plant to resist infection. Analyses show the *Glycine max* PGIP11 (*GmPGIP11*) is expressed within a root cell that is parasitized by the pathogenic nematode *Heterodera glycines*, the soybean cyst nematode (SCN), but while undergoing a defence response that leads to its demise. Transgenic experiments show *GmPGIP11* overexpression leads to a successful defence response, while the overexpression of a related *G. max* PGIP, *GmPGIP1* does not, indicating a level of specificity. The analyses presented here have identified PGIPs from 51 additional studied proteomes, many of agricultural importance. The analyses include the computational identification of signal peptides and their cleavage sites, *O*-, and *N*-glycosylation. Artificial intelligence analyses determine the location where the processed protein localize. The identified PGIPs are presented as a tool base from which functional transgenics can be performed to determine whether they may have a role in plant-pathogen interactions.

Specifications Table

**Table:** 

Subject	Biological sciences
Specific subject area	Omics: Genomics
Data format	Raw, Analysed, Filtered
Type of data	Table, Figure
Data collection	**Analysed proteomes** The 11 *G. max* PGIP protein sequences are used in Basic Local Alignment Search Tool program (BLAST) searches of the proteomes (BLASTP) using the default parameters at Phytozome (http://www.phytozome.net/). The identified PGIP proteins are compiled using a Bitscore of 140 as a cutoff. To identify the PGIP proteins, each of the 11 *G. max* PGIP protein sequences are queried into the studied proteomes. The individual queries for *Gm*PGIP1 through *Gm*PGIP11 are stored in individual tabs in Excel. Then, the PGIPs that have Bitscores of 140 or higher are compiled for all of the queries for the individual *Gm*PGIPs. The duplicate PGIPs then are removed in Excel. The analysis results in a list of PGIP proteins that include the products of alternate splicing so the numbers in some cases are higher than the numbers of genes in some genomes. **Signal peptide prediction** Signal peptide prediction is done using SignalP 6.0. The default parameters are used. ***O*-glycosylation determination** *O*-glycosylation is determined using NetOGlyc - 4.0. The parameters are set on default. ***N*-glycosylation determination** *N*-glycosylation is determined using NetNGlyc - 1.0. The parameters are on set default. **Protein alignment** Protein alignment is performed using CLUSTAL Omega, CLUSTAL O(1.2.4) multiple sequence alignment. The analysis is performed using default parameters. **Artificial intelligence** Prediction of eukaryotic protein subcellular localization using deep learning is done using DeepLoc-1.0 in default settings.
Data source location	Data obtained from Phytozome (http://www.phytozome.net/)
Data accessibility	Direct URL to data: https://data.mendeley.com/datasets/66r9pkckjz/1

## 1 Value of the Data

• Why are these data valuable?

Plants have a 2-tiered defense platform allowing them to defend themselves from pathogens [1]. The plant recognizes epitopes produced directly or indirectly as a consequence of the plant-pathogen interaction [1]. The epitopes are collectively called pathogen activated molecular patterns (PAMPs) acting within a 2-tiered defense system involving PAMP (pattern) triggered immunity (PTI) and effector triggered immunity (ETI) [1].

Plant cell walls are an important barrier to pathogen infection. Up to 60% of the cell wall pectic moieties of dicot and nongraminaceous monocot primary cell walls are homogalacturonans (HGs), the major component of the middle lamella [2]. Pathogen polygalacturonases (PGs) are effective in facilitating pathogenicity because they break down cell wall polymers, permitting infection [3]. The study presented here is valuable to those interested in understanding plant defence, the evolutionary processes behind defence processes, cell signalling, and an understanding of basic cellular processes.

• Who can benefit from these data?

In order to impede pathogen PGs, plants secrete polygalacturonase inhibiting proteins (PGIPs). PGIPs have a bimodal function. Firstly, PGIPs directly inhibit PGs. Secondly, PG activity leads to oligogalacturonide (OG) accumulation, eliciting a defence response [4]. Therefore, PGIPs deactivate the pathogen effector while also leading to the production and amplification of a signalling cascade. This signal cascade further impairs the pathogen, leading to their demise. For example, a *Beta vulgaris* (sugar beet) *PGIP*, when expressed in *Nicotiana benthamiana*, limits the pathogenicity of *Rhizoctonia solani*, *Fusarium solani,* and *Botrytis cinerea* whose pathogenicity is normally driven by their PGs [5]. Previous work on *G. max PGIP*s (*GmPGIP*s) have functionally examined them [6], benefitting stakeholders interested in the development of pathogen-resistant crops, including *Beta vulgaris* ssp. vulgaris (sugar beet). Novel signalling events can also be determined through the study presented here.

• How can these data be reused by other researchers?

Using 11 *G. max* PGIP protein sequences, the analysis presented here extracts the PGIPs that exist in 51 additional genomes of other important crops and other flowering plants. Analyses determine whether the 469 proteins have signal sequences, compatible with them being secreted proteins, a cleavage site, and whether they are *O*- and/or *N*-glycosylated. Artificial Intelligence analyses show which cellular locale the proteins can be expected to exist, complementing recent transgenic studies of the *GmPGIP11*. The provided analysis and accompanying data can be re-used to basic aspects of plant cell biology and generate pathogen-resistance in a wide spectrum of agriculturally-important crops. The evolution of defence and signalling processes can also be examined.

## 2 Data Description

A total of 469 proteins obtained from Phytozome, not including *G. max*, are analysed, spanning 51 proteomes (Table 1, Supplemental Data File 1) [7]. The proteins annotated as probable PGIPs pass a cutoff between 300 and 399 AAs, within the range of known PGIPs. Among them, 394 putative PGIPs are between 300 and 399 AAs (84%). Among the 51 proteomes, 45 (9.6%) are shorter than 300 AAs. Furthermore, 30 (6.4%) proteins annotated as PGIPs are identified as being 400 AAs or larger. LRRS have a low overall homology based on the LRR composition. For example, *Bv*PGIP6 (EL10Ac4g07809.1) is annotated as being 1,383 AAs. When a BLASTP analysis is run, it is shown to be homologous to the 1,249 AA GASSHO1 (GSO1) (OAO97463.1) as well as the 332 AA polygalacturonase inhibiting protein 1 (AAM65836.1). A re-annotation of the PGIPs is beyond the scope of the study.

**Table 1:** The proteomes under study.

Genome number	Species	genome	Order	Family
1	*Amborella trichopoda*	Amborella trichopoda v1.0	**Amborellales**	**Amborellaceae**
2	*Amaranthus hypochondriacus*	Amaranthus hypochondriacus v2.1	Caryophyllales	Amaranthaceae
3	*Beta vulgaris*	Beta vulgaris EL10_1.0	Caryophyllales	Amaranthaceae
4	*Chenopodium quinoa*	Chenopodium quinoa v1.0	Caryophyllales	Amaranthaceae
5	*Spinacia oleracea*	Spinacia oleracea Spov3	Caryophyllales	Amaranthaceae
6	*Coffea arabica*	Coffea arabica v0.5	Gentianales	Rubiaceae
7	*Daucus carota*	Daucus carota v2.0	Apiales	Apiaceae
8	*Helianthus annuus*	Helianthus annuus r1.2	Asterales	Asteraceae
9	*Lactuca sativa*	Lactuca sativa V8	Asterales	Asteraceae
10	*Mimulus guttatus*	M.guttatus_TOL v5.0	Lamiales	Phrymaceae
11	*Olea europaea*	Olea europaea v1.0	Lamiales	Oleaceae
12	*Solanum lycopersicum*	Solanum lycopersicum ITAG4.0	Solanales	Solanaceae
13	*Solanum tuberosum*	Solanum tuberosum v6.1	Solanales	Solanaceae
14	*Vaccinium darrowii*	V.darrowii v1.2	Ericales	Ericaceae
15	*Eucalyptus grandis*	Eucalyptus grandis v2.0	Myrtales	Myrtaceae
16	*Vitis vinifera*	Vitis vinifera v2.1	Vitales	Vitaceae
17	*Arachis hypogaea*	Arachis hypogaea v1.0	Fabales	Fabaceae
18	*Castanea dentata*	Castanea dentata v1.1	Fagales	Fagaceae
19	*Cicer arietinum*	Cicer arietinum v1.0	Fabales	Fabaceae
20	*Cucumis sativus*	Cucumis sativus v1.0	Cucurbitales	Cucurbitaceae
21	*Fragaria vesca*	Fragaria vesca v4.0.a2	Rosales	Rosaceae
22	*Malus domestica*	Malus domestica v1.1	Rosales	Rosaceae
23	*Medicago truncatula*	Medicago truncatula Mt4.0v1	Fabales	Fabaceae
24	*Phaseolus vulgaris*	Phaseolus vulgaris v2.1	Fabales	Fabaceae
25	*Prunus persica*	Prunus persica v2.1	Rosales	Rosaceae
26	*Quercus rubra*	Quercus rubra v2.1	Fagales	Fagaceae
27	*Trifolium pratense*	Trifolium pratense v2	Fabales	Fabaceae
28	*Carya illinoinensis*	Carya illinoinensis v1.1	Fabales	Juglandaceae
29	*Vigna unguiculata*	Vigna unguiculata v1.2	Fabales	Fabaceae
30	*Linum usitatissimum*	Linum usitatissimum v1.0	Malpighiales	Linaceae
31	*Manihot esculenta*	Manihot esculenta v8.1	Malpighiales	Euphorbiaceae
32	*Carica papaya*	Carica papaya ASGPBv0.4	Brassicales	Caricaceae
33	*Theobroma cacao*	Theobroma cacao v2.1	Malvales	Malvaceae
34	*Arabidopsis thaliana*	Arabidopsis thaliana TAIR10	Brassicales	Brassicaceae
35	*Schrenkiella parvula*	Schrenkiella parvula v2.2	Brassicales	Brassicaceae
36	*Brassica oleracea capitata*	Brassica oleracea capitata v1.0	Brassicales	Brassicaceae
37	*Brassica rapa*	Brassica rapa FPsc v1.3	Brassicales	Brassicaceae
38	*Sinapis alba*	Sinapis alba v3.1	Brassicales	Brassicaceae
39	*Gossypium hirsutum*	Gossypium hirsutum v2.1	Malvales	Malvaceae
40	*Citrus sinensis*	Citrus sinensis v1.1	Sapindales	Rutaceae
41	*Ananas comosus*	Ananas comosus v3	Poales	Bromeliaceae
42	*Dioscorea alata*	Dioscorea alata v2.1	Dioscoreales	Dioscoreaceae
43	*Musa acuminata*	Musa acuminata v1	Zingiberales	Musaceae
44	*Hordeum vulgare*	Hordeum vulgare r1	Poales	Poaceae
45	*Oryza sativa*	Oryza sativa v7.0	Poales	Poaceae
46	*Triticum aestivum*	Triticum aestivum v2.2	Poales	Poaceae
47	*Brachypodium distachyon*	Brachypodium distachyon v3.2	Poales	Poaceae
48	*Miscanthus sinensis*	Miscanthus sinensis v7.1	Poales	Poaceae
49	*Sorghum bicolor*	Sorghum bicolor v3.1.1	Poales	Poaceae
50	*Zea mays*	Zea mays RefGen_V4	Poales	Poaceae
51	*Panicum hallii*	Panicum hallii v3.2	Poales	Poaceae
52	*Glycine max*	G.max Wm82.a2.v1	Fabales	Fabaceae

### 2.1 Signal peptide prediction

Signal peptide prediction is performed to determine whether the identified 469 proteins exhibiting homology to PGIP have characteristics of secreted proteins (Supplemental Data File 2). The protein sequences are imported into SignalP 6.0 [8,9]. The number of putative PGIPs with predicted signal peptides are identified (Table 2; Supplemental Data File 3).

**Table 2:** The identified PGIPs.

Genome number	Species	genome	PGIP proteins
1	*Amborella trichopoda*	Amborella trichopoda v1.0	2
2	*Amaranthus hypochondriacus*	Amaranthus hypochondriacus v2.1	6
3	*Beta vulgaris*	Beta vulgaris EL10_1.0	9
4	*Chenopodium quinoa*	Chenopodium quinoa v1.0	22
5	*Spinacia oleracea*	Spinacia oleracea Spov3	6
6	*Coffea arabica*	Coffea arabica v0.5	15
7	*Daucus carota*	Daucus carota v2.0	9
8	*Helianthus annuus*	Helianthus annuus r1.2	8
9	*Lactuca sativa*	Lactuca sativa V8	13
10	*Mimulus guttatus*	M.guttatus_TOL v5.0	10
11	*Olea europaea*	Olea europaea v1.0	5
12	*Solanum lycopersicum*	Solanum lycopersicum ITAG4.0	5
13	*Solanum tuberosum*	Solanum tuberosum v6.1	3
14	*Eucalyptus grandis*	Eucalyptus grandis v2.0	9
15	*Vitis vinifera*	Vitis vinifera v2.1	4
16	*Arachis hypogaea*	Arachis hypogaea v1.0	17
17	*Castanea dentata*	Castanea dentata v1.1	5
18	*Cicer arietinum*	Cicer arietinum v1.0	8
19	*Cucumis sativus*	Cucumis sativus v1.0	3
20	*Fragaria vesca*	Fragaria vesca v4.0.a2	4
21	*Malus domestica*	Malus domestica v1.1	8
22	*Medicago truncatula*	Medicago truncatula Mt4.0v1	23
23	*Phaseolus vulgaris*	Phaseolus vulgaris v2.1	9
24	*Prunus persica*	Prunus persica v2.1	5
25	*Quercus rubra*	Quercus rubra v2.1	7
26	*Trifolium pratense*	Trifolium pratense v2	16
27	*Carya illinoinensis*	Carya illinoinensis v1.1	3
28	*Vigna unguiculata*	Vigna unguiculata v1.2	10
29	*Linum usitatissimum*	Linum usitatissimum v1.0	9
30	*Manihot esculenta*	Manihot esculenta v8.1	6
31	*Carica papaya*	Carica papaya ASGPBv0.4	3
32	*Theobroma cacao*	Theobroma cacao v2.1	4
33	*Arabidopsis thaliana*	Arabidopsis thaliana TAIR10	6
34	*Schrenkiella parvula*	Schrenkiella parvula v2.2	6
35	*Brassica oleracea capitata*	Brassica oleracea capitata v1.0	19
36	*Brassica rapa*	Brassica rapa FPsc v1.3	19
37	*Sinapis alba*	Sinapis alba v3.1	26
38	*Gossypium hirsutum*	Gossypium hirsutum v2.1	6
39	*Citrus sinensis*	Citrus sinensis v1.1	4
40	*Ananas comosus*	Ananas comosus v3	5
41	*Dioscorea alata*	Dioscorea alata v2.1	9
42	*Musa acuminata*	Musa acuminata v1	5
43	*Hordeum vulgare*	Hordeum vulgare r1	13
44	*Oryza sativa*	Oryza sativa v7.0	9
45	*Triticum aestivum*	Triticum aestivum v2.2	19
46	*Brachypodium distachyon*	Brachypodium distachyon v3.2	7
47	*Miscanthus sinensis*	Miscanthus sinensis v7.1	13
48	*Sorghum bicolor*	Sorghum bicolor v3.1.1	8
49	*Zea mays*	Zea mays RefGen_V4	9
50	*Panicum hallii*	Panicum hallii v3.2	7
51	*Vaccinium darrowii*	V.darrowii v1.2	13
TOTAL			469

### 2.2 Comparison of *O*- and *N*-glycosylation of *Gm*PGIPs

A companion analysis demonstrates that *Gm*PGIP11 but not *Gm*PGIP1 functions in the defence response that *G. max* has toward *H. glycines* parasitism. A comparative analysis of *G. max* PGIPs is undertaken to determine whether *O*- and/or *N*-glycosylation could be correlated to these differences. The *O*-glycosylation analysis demonstrates that while *Gm*PGIP1 is *O*-glycosylated, *Gm*PGIP11 is not (Table 3; Supplemental Data File 4).

**Table 3:** The signal peptide prediction, *O*-, *N*-glycosylation prediction, cellular location prediction.

Species/PGIP protein	SP	O glyc	N-glyc	Location	Species/PGIP protein	SP	O glyc	N-glyc	Location
*Amborella trichopoda*					*Vigna unguiculata*				
AmtPGIP1	y	Y	y	Extracellular	VuPGIP1	y	y	y	Extracellular
AmtPGIP2	y	Y	y	Extracellular	VuPGIP2	y	n	y	Extracellular
*Amaranthus hypochondriacus*					VuPGIP3	y	n	y	Extracellular
AhyPGIP1	y	N	y	Extracellular	VuPGIP4	y	n	y	Extracellular
AhyPGIP2	y	N	y	Extracellular	VuPGIP5	y	n	y	Extracellular
AhyPGIP3	y	N	y	Extracellular	VuPGIP6	n	y	y	Extracellular
AhyPGIP4	n	N	y	Cytoplasm	VuPGIP7	y	n	y	Extracellular
AhyPGIP5	y	Y	y	Extracellular	VuPGIP8	y	n	y	Extracellular
AhyPGIP6	n	N	y	Extracellular	VuPGIP9	y	n	y	Extracellular
*Beta vulgaris*					VuPGIP10	y	n	y	Extracellular
BvPGIP1	y	Y	y	Extracellular	*Linum usitatissimum*				
BvPGIP2	y	Y	y	Extracellular	LuPGIP1	y	y	y	Extracellular
BvPGIP3	y	Y	y	Extracellular	LuPGIP2	y	y	y	Extracellular
BvPGIP4	y	Y	y	Extracellular	LuPGIP3	y	y	y	Extracellular
BvPGIP5	y	N	y	Extracellular	LuPGIP4	y	n	y	Extracellular
BvPGIP6	y	Y	y	Extracellular	LuPGIP5	y	y	y	Extracellular
BvPGIP7	y	N	y	Extracellular	LuPGIP6	y	n	y	Extracellular
BvPGIP8	n	Y	y	Extracellular	LuPGIP7	n	n	y	Cytoplasm
BvPGIP9	y	Y	y	Extracellular	LuPGIP8	y	y	y	Extracellular
*Chenopodium quinoa*					LuPGIP9	y	y	y	Extracellular
CqPGIP1	n	Y	y	Extracellular	*Manihot esculenta*				
CqPGIP2	y	Y	y	Extracellular	MePGIP1	y	n	y	Extracellular
CqPGIP3	y	Y	y	Extracellular	MePGIP2	y	n	y	Extracellular
CqPGIP4	y	Y	y	Extracellular	MePGIP3	y	n	y	Extracellular
CqPGIP5	y	N	y	Extracellular	MePGIP4	y	n	y	Extracellular
CqPGIP6	y	Y	y	Extracellular	MePGIP_5	n	y	y	Extracellular
CqPGIP7	y	N	y	Extracellular	MePGIP6	y	y	y	Celll membrane
CqPGIP8	n	N	y	Extracellular	*Carica papaya*				
CqPGIP9	n	N	y	Extracellular	CpPGIP1	y	n	y	Extracellular
CqPGIP10	y	N	y	Extracellular	CpPGIP2	y	n	y	Extracellular
CqPGIP11	n	Y	y	Nucleus	CpPGIP3	y	n	y	Extracellular
CqPGIP12	n	N	y	Cytoplasm	*Theobroma cacao*				
CqPGIP13	n	N	y	Cytoplasm	TcPGIP1	y	n	y	Extracellular
CqPGIP14	y	N	y	Extracellular	TcPGIP2	y	n	y	Extracellular
CqPGIP15	n	N	y	Extracellular	TcPGIP3	y	y	y	Extracellular
CqPGIP16	n	N	y	Nucleus	TcPGIP4	y	n	y	Extracellular
CqPGIP17	y	N	y	Extracellular	*Arabidopsis thaliana*				
CqPGIP18	y	N	y	Extracellular	AtPGIP1	y	n	y	Extracellular
CqPGIP19	y	Y	y	Lysosome	AtPGIP2	y	y	y	Extracellular
CqPGIP20	n	N	y	Extracellular	AtPGIP3	y	y	y	Extracellular
CqPGIP21	n	N	y	Extracellular	AtPGIP4	y	y	n	Extracellular
CqPGIP22	y	Y	y	Extracellular	AtPGIP5	y	n	y	Extracellular
*Spinacia oleracea*					AtPGIP6	y	n	y	Extracellular
SoPGIP1	y	Y	y	Extracellular	*Schrenkiella parvula*				
SoPGIP2	y	Y	y	Extracellular	SpPGIP1	y	n	y	Extracellular
SoPGIP3	n	Y	y	Cytoplasm	SpPGIP2	y	n	y	Extracellular
SoPGIP4	y	Y	y	Extracellular	SpPGIP3	y	y	y	Extracellular
SoPGIP5	y	N	y	Extracellular	SpPGIP4	y	n	y	Extracellular
SoPGIP6	y	N	n	Extracellular	SpPGIP5	y	n	y	Extracellular
*Coffea Arabica*					SpPGIP6	y	y	y	Extracellular
CaPGIP1	y	Y	y	Extracellular	*Brassica oleracea capitata*				
CaPGIP2	y	N	y	Extracellular	BoPGIP1	y	n	y	Extracellular
CaPGIP3	y	Y	y	Extracellular	BoPGIP2	n	y	y	Extracellular
CaPGIP4	y	Y	y	Extracellular	BoPGIP3	y	n	y	Extracellular
CaPGIP5	y	Y	y	Extracellular	BoPGIP4	y	y	y	Extracellular
CaPGIP6	n	N	y	Cytoplasm	BoPGIP5	y	y	y	Extracellular
CaPGIP7	n	Y	y	Cytoplasm	BoPGIP6	n	n	y	Extracellular
CaPGIP8	y	N	y	Extracellular	BoPGIP7	n	n	y	Extracellular
CaPGIP9	y	Y	n	Extracellular	BoPGIP8	y	n	y	Extracellular
CaPGIP10	y	N	y	Extracellular	BoPGIP9	y	n	y	Extracellular
CaPGIP11	y	N	y	Extracellular	BoPGIP10	y	y	y	Extracellular
CaPGIP12	y	N	y	Extracellular	BoPGIP11	n	n	y	Cytoplasm
CaPGIP13	y	N	y	Extracellular	BoPGIP12	y	y	y	Extracellular
CaPGIP14	y	N	y	Extracellular	BoPGIP13	y	n	y	Extracellular
CaPGIP15	y	N	y	Extracellular	BoPGIP14	y	y	y	Extracellular
*Daucus carota*					BoPGIP15	y	n	y	Extracellular
DcPGIP1	y	N	y	Extracellular	BoPGIP16	y	n	y	Extracellular
DcPGIP2	y	N	y	Extracellular	BoPGIP17	y	n	n	Extracellular
DcPGIP3	y	N	y	Extracellular	BoPGIP18	y	y	y	Extracellular
DcPGIP4	n	N	y	Extracellular	BoPGIP19	y	n	y	Extracellular
DcPGIP5	y	N	y	Extracellular	*Brassica rapa*				
DcPGIP6	y	Y	y	Extracellular	BrPGIP1	y	n	y	Extracellular
DcPGIP7	y	N	n	Extracellular	BrPGIP2	y	n	y	Extracellular
DcPGIP8	n	N	y	Not find	BrPGIP3	y	n	y	Extracellular
DcPGIP9	y	Y	y	Extracellular	BrPGIP4	y	n	y	Extracellular
*Helianthus annuus*					BrPGIP5	y	n	y	Extracellular
HaPGIP1	y	Y	y	Extracellular	BrPGIP6	y	y	y	Extracellular
HaPGIP2	y	Y	y	Extracellular	BrPGIP7	y	n	y	Extracellular
HaPGIP3	y	N	y	Extracellular	BrPGIP8	y	y	y	Extracellular
HaPGIP4	y	Y	y	Extracellular	BrPGIP9	y	n	y	Extracellular
HaPGIP5	y	N	y	Extracellular	BrPGIP10	y	y	y	Extracellular
HaPGIP6	y	N	y	Extracellular	BrPGIP11	y	y	y	Extracellular
HaPGIP7	y	N	y	Extracellular	BrPGIP12	y	n	y	Extracellular
HaPGIP8	y	N	y	Extracellular	BrPGIP13	y	y	y	Extracellular
*Lactuca sativa*					BrPGIP14	y	y	y	Extracellular
LsPGIP1	y	Y	y	Extracellular	BrPGIP15	y	n	y	Extracellular
LsPGIP2	y	Y	y	Extracellular	BrPGIP16	y	n	n	Extracellular
LsPGIP3	y	Y	y	Extracellular	BrPGIP17	y	n	y	Extracellular
LsPGIP4	y	N	y	Extracellular	BrPGIP18	y	y	y	Extracellular
LsPGIP5	y	Y	y	Extracellular	BrPGIP19	y	y	y	Extracellular
LsPGIP6	y	N	y	Extracellular	*Sinapis alba*				
LsPGIP7	n	N	y	Cytoplasm	SaPGIP1	y	n	y	Extracellular
LsPGIP8	y	N	y	Extracellular	SaPGIP2	y	n	y	Extracellular
LsPGIP9	y	N	y	Extracellular	SaPGIP3	y	n	y	Extracellular
LsPGIP10	y	N	y	Extracellular	SaPGIP4	y	n	y	Extracellular
LsPGIP11	y	N	y	Extracellular	SaPGIP5	y	n	y	Extracellular
LsPGIP12	y	N	y	Extracellular	SaPGIP6	y	y	y	Extracellular
LsPGIP13	y	N	y	Extracellular	SaPGIP7	n	y	y	Nucleus
*Mimulus guttatus*					SaPGIP8	y	y	y	Extracellular
MgPGIP1	y	N	y	Extracellular	SaPGIP9	y	y	y	Extracellular
MgPGIP2	y	Y	y	Extracellular	SaPGIP10	y	n	y	Extracellular
MgPGIP3	y	N	y	Extracellular	SaPGIP11	y	y	y	Extracellular
MgPGIP4	y	Y	y	Extracellular	SaPGIP12	y	n	y	Extracellular
MgPGIP5	y	Y	y	Extracellular	SaPGIP13	y	y	y	Extracellular
MgPGIP6	y	Y	y	Extracellular	SaPGIP14	y	n	y	Extracellular
MgPGIP7	n	Y	y	Cytoplasm	SaPGIP15	y	n	y	Extracellular
MgPGIP8	n	Y	y	Cytoplasm	SaPGIP16	y	n	y	Extracellular
MgPGIP9	y	Y	y	Extracellular	SaPGIP17	y	n	y	Extracellular
MgPGIP10	y	Y	y	Cell membrane	SaPGIP18	y	n	y	Extracellular
*Olea europaea*		N	n		SaPGIP19	y	n	y	Extracellular
OePGIP1	y	N	y	Extracellular	SaPGIP20	y	y	n	Extracellular
OePGIP2	y	N	y	Extracellular	SaPGIP21	n	n	y	Extracellular
OePGIP3	y	N	y	Extracellular	SaPGIP22	n	n	y	Lysosome
OePGIP4	n	N	y	Nucleus	SaPGIP23	y	n	y	Extracellular
OePGIP5	n	Y	y	Cell membrane	SaPGIP24	y	n	y	Extracellular
*Solanum lycopersicum*					SaPGIP25	y	n	y	Extracellular
SlPGIP1	y	N	y	Extracellular	SaPGIP26	y	y	n	Extracellular
SlPGIP2	y	N	y	Extracellular	*Gossypium hirsutum*				
SlPGIP3	y	N	y	Extracellular	GhPGIP1	y	n	y	Extracellular
SlPGIP4	y	Y	y	Extracellular	GhPGIP2	y	y	y	Extracellular
SlPGIP5	y	N	y	Extracellular	GhPGIP3	y	n	y	Extracellular
*Solanum tuberosum*					GhPGIP4	y	y	y	Extracellular
StPGIP1	y	N	y	Extracellular	GhPGIP5	y	n	y	Extracellular
StPGIP2	y	Y	y	Extracellular	GhPGIP6	y	n	y	Extracellular
StPGIP3	y	Y	y	Extracellular	*Citrus sinensis*				
*Eucalyptus grandis*					CsPGIP1	y	n	y	Extracellular
EgPGIP1	n	N	y	Nucleus	CsPGIP2	y	n	y	Extracellular
EgPGIP2	n	Y	y	Cytoplasm	CsPGIP3	y	y	y	Extracellular
EgPGIP3	y	N	y	Extracellular	CsPGIP4	y	n	y	Extracellular
EgPGIP4	n	N	y	Extracellular	*Ananas comosus*				
EgPGIP5	n	N	y	Extracellular	AcPGIP1	y	y	y	Extracellular
EgPGIP6	y	N	y	Extracellular	AcPGIP2	y	y	y	Extracellular
EgPGIP7	y	N	y	Extracellular	AcPGIP3	y	n	y	Extracellular
EgPGIP8	y	Y	y	Extracellular	AcPGIP4	y	n	y	Extracellular
EgPGIP9	y	Y	y	Extracellular	AcPGIP5	y	n	y	Extracellular
*Vitis vinifera*					*Dioscorea alata*				
VvPGIP1	y	N	y	Extracellular	DaPGIP1	y	y	y	Extracellular
VvPGIP2	y	N	y	Extracellular	DaPGIP2	y	n	y	Extracellular
VvPGIP3	y	N	y	Extracellular	DaPGIP3	y	y	y	Extracellular
VvPGIP4	y	N	y	Extracellular	DaPGIP4	y	n	y	Extracellular
*Arachis hypogaea*					DaPGIP5	n	y	y	Extracellular
AhPGIP1	y	N	y	Extracellular	DaPGIP6	y	n	y	Extracellular
AhPGIP2	y	N	y	Extracellular	DaPGIP7	y	y	y	Extracellular
AhPGIP3	y	N	y	Extracellular	DaPGIP8	y	y	y	Extracellular
AhPGIP4	y	N	y	Extracellular	DaPGIP9	y	y	y	Extracellular
AhPGIP5	y	Y	y	Extracellular	*Musa acuminata*				
AhPGIP6	y	Y	y	Extracellular	MaPGIP1	y	y	y	Extracellular
AhPGIP7	y	N	y	Extracellular	MaPGIP2	y	n	y	Extracellular
AhPGIP8	y	N	y	Extracellular	MaPGIP3	n	y	y	Cytoplasm
AhPGIP9	y	Y	y	Extracellular	MaPGIP4	y	y	y	Extracellular
AhPGIP10	y	Y	y	Extracellular	MaPGIP5	n	y	y	Extracellular
AhPGIP11	y	Y	y	Extracellular	*Hordeum vulgare*				
AhPGIP12	y	Y	y	Extracellular	HvPGIP1	y	y	y	Extracellular
AhPGIP13	y	Y	n	Extracellular	HvPGIP2	y	y	y	Extracellular
AhPGIP14	y	Y	n	Extracellular	HvPGIP3	n	y	y	Extracellular
AhPGIP15	n	Y	y	Cytoplasm	HvPGIP4	y	y	y	Extracellular
AhPGIP16	n	Y	y	Cytoplasm	HvPGIP5	y	y	y	Extracellular
AhPGIP17	y	Y	y	Extracellular	HvPGIP6	y	y	y	Extracellular
*Castanea dentate*					HvPGIP7	y	y	y	Extracellular
CdPGIP1	y	Y	y	Extracellular	HvPGIP8	n	y	y	Extracellular
CdPGIP2	y	N	y	Extracellular	HvPGIP9	n	y	y	Cytoplasm
CdPGIP3	y	N	y	Extracellular	HvPGIP10	y	y	y	Extracellular
CdPGIP4	y	Y	y	Extracellular	HvPGIP11	y	y	y	Extracellular
CdPGIP5	n	N	y	Lysosome	HvPGIP12	y	y	y	Extracellular
*Cicer arietinum*					HvPGIP13	y	y	y	Extracellular
CiaPGIP1	y	Y	y	Extracellular	*Oryza sativa*				
CiaPGIP2	y	N	y	Extracellular	OsPGIP1	y	y	y	Extracellular
CiaPGIP3	y	N	y	Extracellular	OsPGIP2	y	n	y	Extracellular
CiaPGIP4	n	Y	y	Extracellular	OsPGIP3	n	n	y	Cell membrane
CiaPGIP5	y	Y	y	Extracellular	OsPGIP4	y	y	y	Extracellular
CiaPGIP6	y	Y	y	Extracellular	OsPGIP5	y	y	y	Extracellular
CiaPGIP7	y	N	y	Extracellular	OsPGIP6	y	y	y	Extracellular
CiaPGIP8	y	N	y	Extracellular	OsPGIP7	n	y	y	Nucleus
*Cucumis sativus*					OsPGIP8	y	n	y	Extracellular
CusPGIP1	y	N	y	Extracellular	OsPGIP9	y	y	y	Extracellular
CusPGIP2	y	N	y	Extracellular	*Triticum aestivum*				
CusPGIP3	y	N	y	Extracellular	TaPGIP1	y	y	y	Extracellular
*Fragaria vesca*					TaPGIP2	y	y	y	Extracellular
FvPGIP1	y	Y	y	Extracellular	TaPGIP3	y	y	y	Extracellular
FvPGIP2	y	N	y	Extracellular	TaPGIP4	y	y	y	Extracellular
FvPGIP3	y	Y	y	Extracellular	TaPGIP5	y	y	y	Extracellular
FvPGIP4	y	N	y	Extracellular	TaPGIP6	y	y	y	Extracellular
*Malus domestica*					TaPGIP7	y	n	y	Extracellular
MdPGIP1	y	N	y	Extracellular	TaPGIP8	y	n	y	Extracellular
MdPGIP2	y	Y	y	Extracellular	TaPGIP9	y	y	y	Extracellular
MdPGIP3	n	N	y	Lysosome	TaPGIP10	y	y	y	Extracellular
MdPGIP4	y	N	y	Extracellular	TaPGIP11	y	y	y	Extracellular
MdPGIP5	y	N	y	Extracellular	TaPGIP12	y	y	y	Extracellular
MdPGIP6	y	N	y	Extracellular	TaPGIP13	y	y	y	Extracellular
MdPGIP7	y	Y	y	Extracellular	TaPGIP14	y	y	y	Extracellular
MdPGIP8	y	Y	y	Extracellular	TaPGIP15	y	y	y	Extracellular
*Medicago truncatula*					TaPGIP16	y	y	y	Extracellular
MtPGIP1	y	N	y	Extracellular	TaPGIP17	y	y	y	Extracellular
MtPGIP2	y	Y	y	Extracellular	TaPGIP18	n	n	y	Cytoplasm
MtPGIP3	y	Y	y	Extracellular	TaPGIP19	n	n	y	Cytoplasm
MtPGIP4	y	N	y	Extracellular	*Brachypodium distachyon*				
MtPGIP5	y	Y	y	Extracellular	BdPGIP1	y	y	y	Extracellular
MtPGIP6	y	Y	y	Extracellular	BdPGIP2	y	y	y	Extracellular
MtPGIP7	y	N	y	Extracellular	BdPGIP3	y	y	y	Extracellular
MtPGIP8	y	Y	y	Extracellular	BdPGIP4	y	y	y	Extracellular
MtPGIP9	n	N	y	Not find	BdPGIP5	y	y	y	Extracellular
MtPGIP10	y	Y	y	Extracellular	BdPGIP6	y	y	y	Extracellular
MtPGIP11	y	Y	y	Extracellular	BdPGIP7	y	y	y	Extracellular
MtPGIP12	y	Y	y	Extracellular	*Miscanthus sinensis*				
MtPGIP13	y	Y	y	Extracellular	MsPGIP1	y	n	y	Extracellular
MtPGIP14	y	N	y	Extracellular	MsPGIP2	y	y	y	Extracellular
MtPGIP15	y	Y	y	Extracellular	MsPGIP3	y	y	y	Extracellular
MtPGIP16	y	Y	y	Extracellular	MsPGIP4	y	n	y	Extracellular
MtPGIP17	y	Y	y	Extracellular	MsPGIP5	n	y	y	Extracellular
MtPGIP18	n	Y	y	Chloroplast	MsPGIP6	y	y	y	Extracellular
MtPGIP19	y	Y	y	Extracellular	MsPGIP7	y	y	y	Extracellular
MtPGIP20	y	N	y	Extracellular	MsPGIP8	y	n	y	Extracellular
MtPGIP21	y	Y	y	Extracellular	MsPGIP9	y	n	y	Extracellular
MtPGIP22	y	Y	y	Cell membrane	MsPGIP10	n	y	y	Cytoplasm
MtPGIP23	y	Y	y	Extracellular	MsPGIP11	y	n	y	Extracellular
*Phaseolus vulgaris*					MsPGIP12	n	y	y	Extracellular
PvPGIP1	y	Y	y	Extracellular	MsPGIP13	y	y	y	Extracellular
PvPGIP3	y	Y	y	Extracellular	*Sorghum bicolor*				
PvPGIP4	y	Y	y	Extracellular	SbPGIP1	y	y	y	Extracellular
PvPGIP5	y	Y	y	Extracellular	SbPGIP2	y	y	y	Extracellular
PvPGIP6	y	N	y	Extracellular	SbPGIP3	y	y	y	Extracellular
PvPGIP7	y	N	y	Extracellular	SbPGIP4	y	y	y	Extracellular
PvPGIP8	n	Y	y	Extracellular	SbPGIP5	y	y	n	Extracellular
PvPGIP9	y	N	y	Extracellular	SbPGIP6	y	y	y	Extracellular
*Prunus persica*					SbPGIP7	y	y	y	Extracellular
PpPGIP1	y	N	y	Extracellular	SbPGIP8	y	y	y	Extracellular
PpPGIP2	y	N	y	Extracellular	*Zea mays*				
PpPGIP3	y	N	y	Extracellular	ZmPGIP1	y	y	y	Extracellular
PpPGIP4	y	Y	y	Extracellular	ZmPGIP2	y	y	y	Extracellular
PpPGIP5	y	N	y	Extracellular	ZmPGIP3	y	n	y	Extracellular
*Quercus rubra*					ZmPGIP4	y	y	y	Extracellular
QrPGIP1	y	Y	y	Extracellular	ZmPGIP5	y	y	y	Extracellular
QrPGIP2	y	Y	y	Extracellular	ZmPGIP6	y	y	y	Extracellular
QrPGIP3	y	Y	y	Extracellular	ZmPGIP7	y	y	y	Extracellular
QrPGIP4	y	Y	y	Nucleus	ZmPGIP8	y	y	y	Extracellular
QrPGIP5	y	N	y	Extracellular	ZmPGIP9	y	y	y	Extracellular
QrPGIP6	y	N	y	Extracellular	*Panicum hallii*				
QrPGIP7	y	Y	y	Cytoplasm	PhPGIP1	y	y	y	Extracellular
*Trifolium pretense*					PhPGIP2	y	y	y	Extracellular
TpPGIP1	y	Y	y	Extracellular	PhPGIP3	y	y	y	Extracellular
TpPGIP2	y	Y	y	Extracellular	PhPGIP4	y	y	y	Extracellular
TpPGIP3	y	Y	y	Extracellular	PhPGIP5	y	y	y	Extracellular
TpPGIP4	y	Y	y	Extracellular	PhPGIP6	y	y	y	Extracellular
TpPGIP5	y	Y	y	Extracellular	PhPGIP7	y	y	y	Cell membrane
TpPGIP6	y	N	y	Extracellular	*Vaccinium darrowii*				
TpPGIP7	y	Y	y	Extracellular	VdPGIP1	y	y	y	Extracellular
TpPGIP8	y	N	y	Extracellular	VdPGIP2	y	y	y	Extracellular
TpPGIP9	y	N	y	Extracellular	VdPGIP3	y	y	y	Extracellular
TpPGIP10	y	Y	y	Extracellular	VdPGIP4	y	y	y	Extracellular
TpPGIP11	n	Y	y	Cell membrane	VdPGIP5	y	n	y	Extracellular
TpPGIP12	y	Y	y	Extracellular	VdPGIP6	y	y	y	Extracellular
TpPGIP13	y	N	y	Extracellular	VdPGIP7	y	y	y	Extracellular
TpPGIP14	y	Y	y	Cell membrane	VdPGIP8	y	n	y	Extracellular
TpPGIP15	y	Y	y	Extracellular	VdPGIP9	n	n	y	Cytoplasm
TpPGIP16	n	Y	y	Extracellular	VdPGIP10	y	n	y	Extracellular
*Carya illinoinensis*					VdPGIP11	y	y	y	Extracellular
CiPGIP1	y	Y	n	Extracellular	VdPGIP12	y	y	y	Extracellular
CiPGIP2	y	Y	n	Extracellular	VdPGIP13	n	y	y	Extracellular
CiPGIP3	y	Y	n	Extracellular	*Glycine max*				
					GmPGIP1	y	y	y	Extracellular
					GmPGIP2	n	y	y	Cytoplasm
					GmPGIP3	y	y	y	Extracellular
					GmPGIP4	y	y	y	Extracellular
					GmPGIP5	n	y	y	Nucleus
					GmPGIP6	y	y	y	Extracellular
					GmPGIP7	y	y	y	Extracellular
					GmPGIP8	y	y	y	Extracellular
					GmPGIP9	n	y	y	Cell membrane
					GmPGIP10	y	y	y	Extracellular
					GmPGIP11	y	n	y	Extracellular

**Fig. 1:**
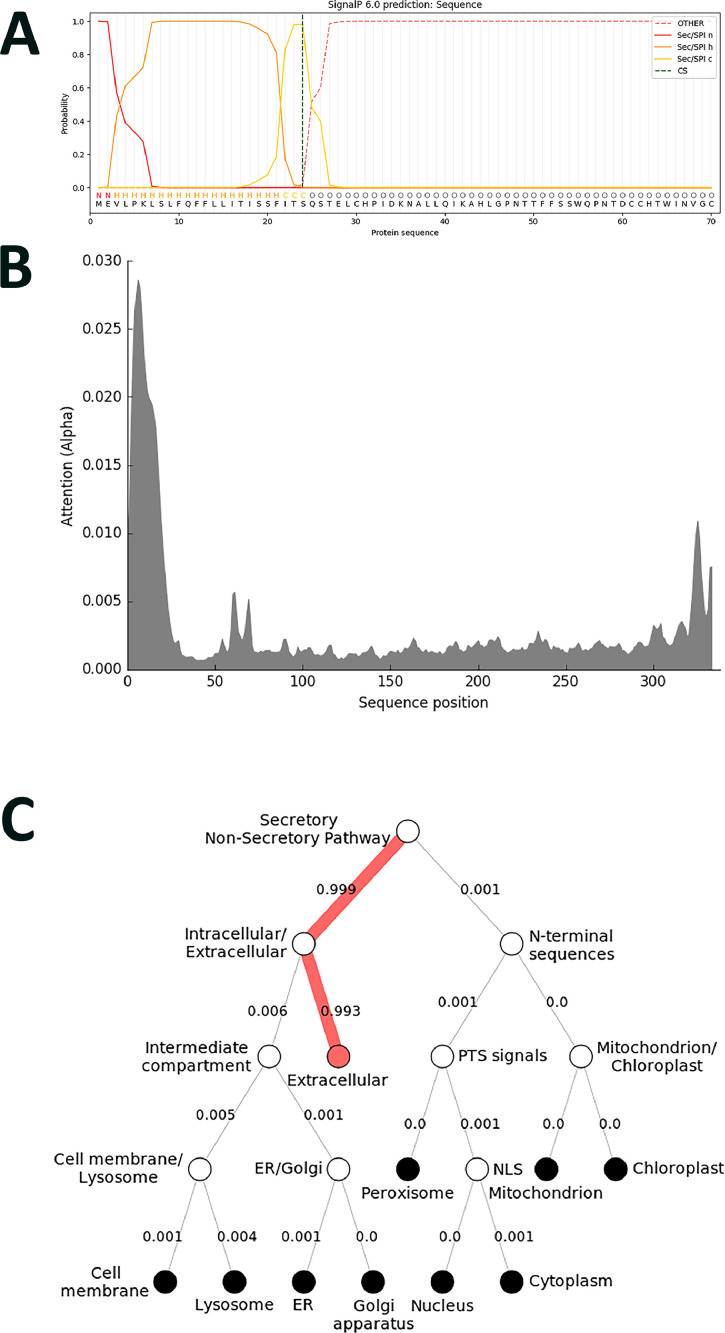
*Beta vulgaris Bv*PGIP4 protein analysis. **A**. Signal peptide prediction. **B**. Amino acid relative importance. **C**. Hierarchical tree, showing the localization likelihood in numerical value: Extracellular, 0.9701; Lysosome/Vacuole, 0.0263; Endoplasmic reticulum, 0.0024; Cell membrane, 0.0007; Cytoplasm, 0.0003; Mitochondrion, 0.0001; Golgi apparatus, 0; Plastid, 0; Nucleus, 0; Peroxisome, 0; Soluble, 0.9994; Membrane, 0.0006. Prediction: Extracellular, Soluble.

In contrast to the above-presented findings both *Gm*PGIP1 and *Gm*PGIP11 are predicted to be *N*-glycosylated. However, some of their predicted N-glycosylation sites are not at homologous aa positions (Fig. 1; Supplemental Data File 5). For example, the NPTT site found in *Gm*PGIP1 and starting at aa position 41 is not identified in *Gm*PGIP11 (Fig.). In contrast, a NLSG site found in *Gm*PGIP11 and starting at aa position 101 is not found in *Gm*PGIP1. Similarly, an NLSG predicted *N*-glycosylation site found in *Gm*PGIP11 and starting at aa position 174 is not found in *Gm*PGIP1 (Fig. 1). Furthermore, an NKTT predicted *N*-glycosylation site found in *Gm*PGIP11 and starting at aa position 258 is not found in *Gm*PGIP1 (Fig. 1). However, *N*-glycosylation sites that are in homologous positions between *Gm*PGIP1 and *Gm*PGIP11 do exist (Fig. 1). *Gm*PGIP1 has a NVSG predicted *N*-glycosylation site starting at aa position 132 while *Gm*PGIP11 has a NVSG predicted *N*-glycosylation site starting at aa position 150 (Fig. 1). Consequently, while experimentation has not proven that these sites are important to the functional differences occurring between *Gm*PGIP1 and *Gm*PGIP11, they are different and provide a basis for future experimentation.

### 2.3 Artificial intelligence

**Fig. 2:**
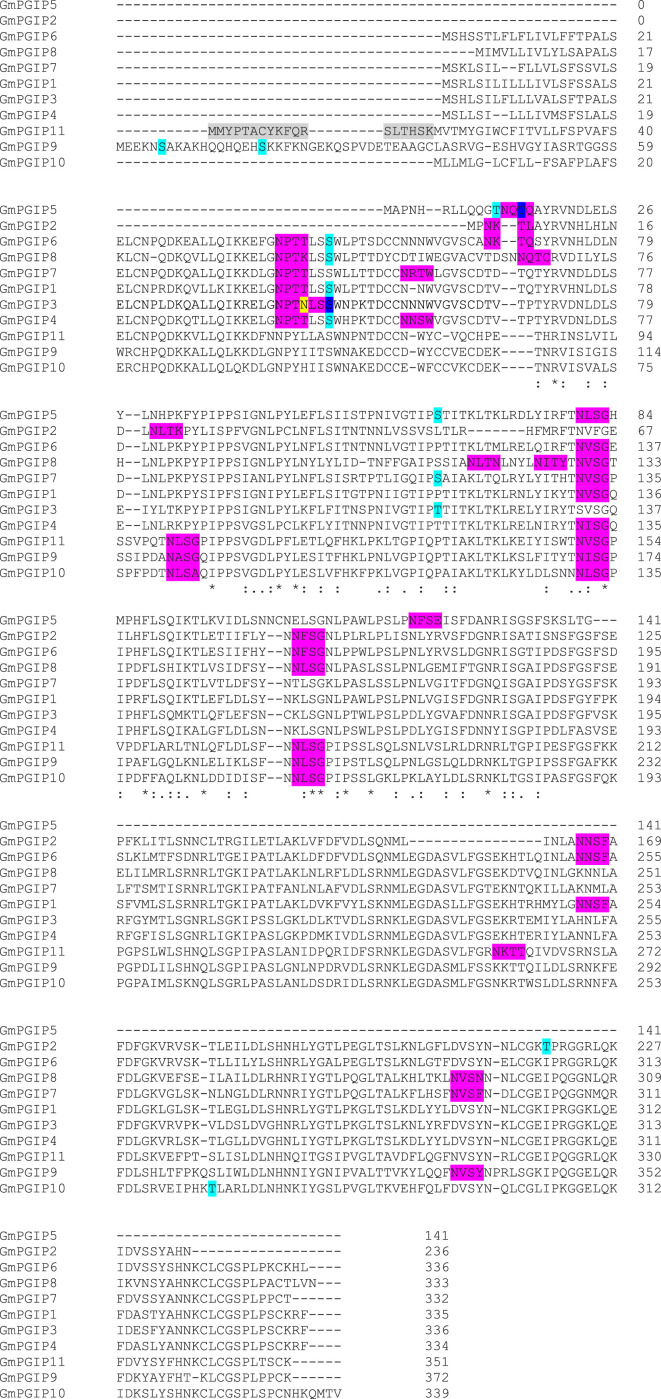
Predicted *O*- and *N*-glycosylation sites of *G. max* PGIP proteins. Cyan, predicted *O*-glycosylation site. Magenta, *N*-glycosylation site. Yellow, an aa that overlaps between two predicted *N*-glycosylation sites. Blue, an aa that overlaps between an *O*- and *N*-glycosylation site. Gray, possible mis-annotated N-terminal sequence.

The 469 identified PGIP proteins spanning the 51 genomes are assessed by artificial intelligence analyses to produce a sequence position file (Supplemental Data File 6). A second file generates a map to the cellular destination where the predicted protein is predicted to function (Table 3; Supplemental Data File 7). An example for *Beta vulgaris Bv*PGIP4, shown to function in defence to various pathogens in *N. tabacum*, is presented (Fig. 2) [5].

The secretion of plant proteins is an important cellular property used for a variety of processes including development and disease resistance [10]. The data presented here is computational support showing PGIPs identified as belonging to taxa positioned at the base of angiosperm evolution are predicted to have signal peptides, have *O-* and/or *N*-glycosylation, and undergo secretion into the apoplast. Further assessment identifies PGIPs from both monocot and dicot lineages with predicted signal peptides and the subcellular or supracellular compartment to which they are targeted [5,11].

### 2.4 Analysed proteomes

The study analyses the proteomes of 51 plants not including *G. max*, many important to agriculture. The 51 proteomes span the base of angiosperm evolution (*A. trichopoda*), a monotypic genus of Amborellaceae and the only member of the Amborellales that has 2 predicted PGIP proteins [12]. Each PGIP is predicted to have signal peptides, experience *O*- and *N*-glycosylation, and undergo secretion into the apoplast. The monocots presented here are represented by *A. comosus, D. alata, M. acuminata, H. vulgare, O. sativa, T. aestivum, B. distachyon, M. sinensis, S. bicolor, Z. mays* and *P. hallii* with the remaining plants belonging to the Eudicots. All of the studied species have at least one putative PGIP that is predicted to have a signal peptide, have *O*- and/or *N*-glycosylation, and are secreted into the apoplast.

Local duplication of plant genes, including PGIPs, results in the generation of genes whose protein products perform an important function in defence [6]. The PGIP proteins identified here also appear to be products of localized gene duplications. Consequently, the identified genes may relate to the birth and death model for PGIPs that is proposed [6]. Possible localized gene duplication is identified from the analysis of the 51 proteomes. Based off the annotations, the analysis, identifying direct tandem duplications for at least one PGIP gene duplication in 29 of the 51 proteomes including *A. hypochondriacus, B. vulgaris, C. quinoa, C. arabica, D. carota, M. guttatus, O. europaea, E. grandis, C. arietinum, M. domestica, M. truncatula, P. vulgaris, P. persica, Q. rubra, V. unguiculata, L. usitatissimum, M. esculenta, T. cacao, A. thaliana, S. parvula, B. oleracea capitata, B. rapa, S. alba, D. alata, V. darrowii, O. sativa, M. sinensis, S. bicolor* and *P. hallii*.

### 2.5 Glycosylation

Computational studies are performed to identify *O*- and/or *N*-glycosylation of the PGIP proteins. Glycosylation is an important feature of proteins that imparts new function and in plants is important in both development and defence [13]. Glycosylation is not a random event and occurs on greater than 50% of eukaryote proteins [14]. *Pyrus communis* (pear) PGIP exhibits heterogeneous glycosylation that relates to pathogen defence [15]. The results presented here provide computational support that plant PGIPs experience glycosylation, broadly.

### 2.6 Sequence alignments identify glycosylation variation that may explain functional differences

Transgenic studies show *Gm*PGIP11 functions in defence to *H. glycines* while *Gm*PGIP1 does not. A computational study analysing the *O*- and *N*-glycosylation sites show that while *Gm*PGIP1 is predicted to be *O*-glycosylated that *Gm*PGIP11 is not. Furthermore, *Gm*PGIP11 has predicted *N*-glycosylation sites that *Gm*PGIP1 does not while *Gm*PGIP1 has predicted *N*-glycosylation sites that are lacking in *Gm*PGIP11. Glycosylation performs important defence roles [16].

### 2.7 Alternate spicing of *PGIP* mRNAs

What has not been presented is the possible importance of alternate RNA splicing in PGIP biology. The analysis presented here identifies 4 proteomes (*L. sativa, P. persica, H. vulgare*, and *T. aestivum*) that are annotated to contain products of alternate splicing. Alternatively spliced transcripts of genes encode transcripts that perform important defence functions, including parasitic nematodes [8].

## 3 Supplemental Data

**Supplemental Data Set 1:** The PGIP accessions obtained by the blast queries according to the described protocol. Supplemental data file 1 - https://data.mendeley.com/datasets/66r9pkckjz/1.

**Supplemental Data Set 2:** The protein sequences obtained from Phytozome. Supplemental data file 2- https://data.mendeley.com/datasets/66r9pkckjz/1.

**Supplemental Data Set 3**: The signal peptide prediction made by SignalP 6.0. Supplemental data file 3- https://data.mendeley.com/datasets/66r9pkckjz/1.

**Supplemental Data Set 4:** The *O*-glycosylation prediction made by NetOGlyc - 4.0. Supplemental data file 4- https://data.mendeley.com/datasets/66r9pkckjz/1.

**Supplemental Data Set 5:** The *N*-glycosylation prediction made by NetNGlyc - 1.0. Supplemental data file 5 - https://data.mendeley.com/datasets/66r9pkckjz/1.

**Supplemental Data Set 6:** The amino acid relative importance predicted by DeepLoc-1.0. Supplemental data file 6- https://data.mendeley.com/datasets/66r9pkckjz/1.

**Supplemental Data Set 7:** The hierarchical trees predicted by DeepLoc-1.0. Supplemental data file 7- https://data.mendeley.com/datasets/66r9pkckjz/1.

## 4 Experimental Design, Materials and Methods

### 4.1 Data access

https://data.mendeley.com/datasets/66r9pkckjz/1.

### 4.2 Analysed proteomes

The 11 *G. max* PGIP protein sequences are used in Basic Local Alignment Search Tool program (BLAST) searches of the proteomes (BLASTP) using the default parameters at Phytozome (http://www.phytozome.net/) [7]. There are 52 total proteomes analysed [7]. The default BLAST parameters are used in querying, including Target type: Proteome; Program: BLASTP-protein query to protein database; Expect (E) threshold: -1; Comparison matrix: BLOcks SUbstitution Matrix (BLOSUM) 62 (BLOSUM62); Word (W) length: default = 3; number of alignments to show: 100 allowing for gaps and filter query, in order that they appear on the BLAST program. Through these analyses it is possible to extract the genomic DNA, transcript, cDNA, protein accessions, their sequences, and gene family members. The analyses also permit the extraction of protein homologs and splice variants from the selected agricultural crops of international importance, those with importance in the U.S., and those important biologically according to [8].

The identified PGIP proteins are compiled using a Bitscore of 140 as a cutoff. To identify the PGIP proteins, each of the 11 *G. max* PGIP protein sequences are queried into the studied proteomes. The individual queries for *Gm*PGIP1 through *Gm*PGIP11 are stored in individual tabs in Excel. Then, the PGIPs that have Bitscores of 140 or higher are compiled for all of the queries for the individual *Gm*PGIPs. The duplicate PGIPs then are removed in Excel. The analysis results in a list of PGIP proteins that include the products of alternate splicing so the numbers in some cases are higher than the numbers of genes in some genomes.

### 4.3 Signal peptide prediction

Signal peptide prediction is done using SignalP 6.0 [9]. SignalP 6.0 is based on protein language models (LMs). The models use information from millions of unannotated protein sequence which are been analysed across all life domains. LMs create logical protein representations capturing their biological structure and properties. SignalP 6.0, thus, predicts additional SP types not possible in earlier iterations of SignalP (e.g., SignalP 5.0) and better extrapolates them to distantly related proteins and ones used to create the model and metagenomic data of unknown origin. SignalP 6.0 also identifies SP subregions. The default parameters are used.

### 4.4 *O*-glycosylation determination

*O*-glycosylation is determined using NetOGlyc - 4.0 [17]. The parameters are set on default. The output format is imported into Excel.

### 4.5 *N*-glycosylation determination

*N*-glycosylation is determined using NetNGlyc - 1.0 set [18]. The parameters are on set default. The output format is imported into Excel.

### 4.6 Protein alignment

Protein alignment is performed using CLUSTAL Omega, CLUSTAL O(1.2.4) multiple sequence alignment [19]. The analysis is performed using default parameters. The output file is imported into MS Word.

### 4.7 Artificial intelligence

Prediction of eukaryotic protein subcellular localization using deep learning is done using DeepLoc-1.0 [20]. The DeepLoc-1.0 analysis determines the importance of a particular amino acid along a protein chain that is relevant for prediction (attention) of its subcellular location and is done in default settings. DeepLoc-1.0 then predicts the subcellular localization of eukaryotic proteins, differentiating between 10 different localizations including the nucleus, cytoplasm, extracellular, mitochondrion, cell membrane, endoplasmic reticulum, (ER) chloroplast, Golgi apparatus, lysosome/vacuole, and peroxisome and is done in default settings. The output of the analysis is presented as a graphic that shows the relative importance of each AA along the polypeptide chain as well as a hierarchical tree that shows where the protein is expected to be located withing a cell [20].

## Limitations

Not applicable.

## Ethics Statement

The authors have read and follow the ethical requirements for publication in Data in Brief and confirm that the current work does not involve human subjects, animal experiments, or any data collected from social media platforms.

## CRediT authorship contribution statement

**Sudha Acharya:** Validation, Formal analysis, Investigation. **Hallie A. Troell:** Validation, Formal analysis, Investigation. **Rebecca L. Billingsley:** Validation, Formal analysis, Investigation. **Katherine S. Lawrence:** Validation, Formal analysis, Investigation, Resources, Writing – original draft, Supervision, Project administration, Funding acquisition. **Daniel S. McKirgan:** Validation, Formal analysis, Investigation. **Nadim W. Alkharouf:** Methodology, Validation, Investigation, Resources, Writing – original draft, Supervision, Project administration. **Vincent P. Klink:** Conceptualization, Methodology, Validation, Investigation, Resources, Writing – original draft, Writing – review & editing, Visualization, Supervision, Project administration, Funding acquisition.

## Data Availability

Supplemental data for Data analysis of polygalacturonase inhibiting proteins (PGIPs) from agriculturally important proteomes (Original data) (Mendeley Data)
